# Indivo: a personally controlled health record for health information exchange and communication

**DOI:** 10.1186/1472-6947-7-25

**Published:** 2007-09-12

**Authors:** Kenneth D Mandl, William W Simons, William CR Crawford, Jonathan M Abbett

**Affiliations:** 1 Children's Hospital Informatics Program at the Harvard-MIT Division of Health Sciences and Technology, Boston, MA, USA; 2 Center for Biomedical Informatics, Harvard Medical School, USA

## Abstract

**Background:**

Personally controlled health records (PCHRs), a subset of personal health records (PHRs), enable a patient to assemble, maintain and manage a secure copy of his or her medical data. Indivo (formerly PING) is an open source, open standards PCHR with an open application programming interface (API).

**Results:**

We describe how the PCHR platform can provide standard building blocks for networked PHR applications. Indivo allows the ready integration of diverse sources of medical data under a patient's control through the use of standards-based communication protocols and APIs for connecting PCHRs to existing and future health information systems.

**Conclusion:**

The strict and transparent personal control model is designed to encourage widespread participation by patients, healthcare providers and institutions, thus creating the ecosystem for development of innovative, consumer-focused healthcare applications.

## Background

Personally controlled health records (PCHRs), [1] a subset of personal health records, [2,3] enable a patient to assemble, maintain and manage a secure copy of his or her medical data [4]. PCHRs are designed on the principle that patients have the right to own and manage copies of their own medical information. PCHRs are complements to, rather than replacements for existing healthcare information management systems. In exercising *control *of their records, individuals decide what data sources populate the record and who is allowed to access or annotate any of the documents contained within the record. Here we describe Indivo [5] (formerly PING [6]), an open source, open standards PCHR with an open application programming interface (API).

Indivo is a specific implementation of a PCHR that is Internet based, provides a World Wide Web interface, and is built to public, open standards. The Indivo software allows institutions to create and administer a PCHR infrastructure that exceeds the requirements of the Health Insurance Portability and Accountability Act (HIPAA) Privacy and Security Rules. As described in Simons et al, Indivo is a three-tier system with a data storage tier, a business logic tier, and a user interface. Indivo's unique implementation of the PCHR concept focuses on complete transparency and high security. All Indivo technical documents, including design concepts and source code, are freely available and accessible on the Internet. Critical to interoperability and adoption, all design concepts, application programming interfaces and document formats are open and public. High security is enforced at all three tiers of the system and is a primary feature.

## Implementation

**Figure 1:**
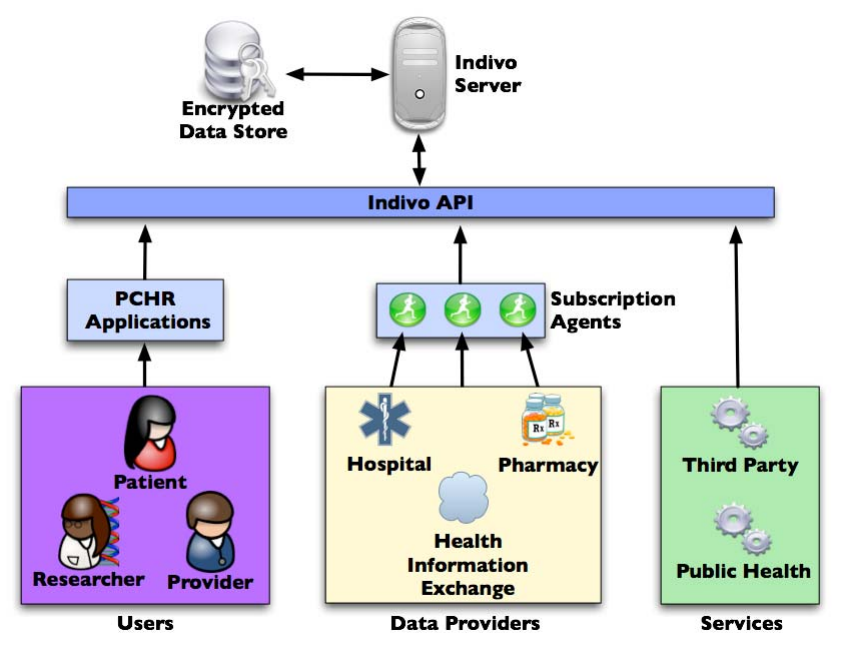
The Indivo architecture demonstrating sources of data – including a health information exchange – and subscription agents (on the left); the three tiered architecture (in the center); and users and services that access the Indivo server (on the right).

Figure 1 shows the conceptual components in Indivo, including the Indivo API, server, encrypted storage, PCHR applications, the subscription agents and data sources, users, and the external services.

The Indivo API is a central and unique feature of the system. The core PCHR functionality, consisting of the ability to aggregate a longitudinal health record from various component data sources, and the ability to share components of an aggregated record with third parties, can be implemented in a variety of ways depending on the originating data sources and the requirements of the user community. The Indivo API allows us to deliver this core functionality in a flexible way, separate from specific implementations of user interfaces or source databases.

### Three tiered architecture

The core of the Indivo platform is the Indivo Server, the "middle" or business logic tier, responsible for managing the set of documents that make up a PCHR record. A document within a PCHR may represent an individual laboratory result, a clinical encounter, allergy, medication, annotation, survey result, or any other discrete piece of relevant healthcare information. The document model was described in Simons et al [6]. The document-based approach provides substantial flexibility, as the server remains agnostic to content and instead acts as a provider of integration and security services. Each Indivo account is implemented as a bundle of documents associated with an individual actor. The Indivo Server makes documents available to client applications via the Indivo API, and determines which documents are available to which users.

The Indivo Server has two classes of security policies. The first is institutionally-oriented, server-based and explicitly permits or denies certain actions. For example, an institutional policy may only allow users assigned a particular role to create new accounts. The second policy type is user-based, enabling a patient to indicate which other users have particular privileges on specific portions of her record. These personal policies are enforced by the Indivo Server along with the institutional policies. For example, users can choose to restrict access to individual documents within their PCHR, or to classes of documents within the PCHR. The server resolves conflicts between user and institutional policies by favoring the strictest policy. That is, if an institutional policy permits a particular action, and the user policy denies it, the action will be denied. Since personal control is a paramount feature, there are only rare instances where the institutional policy to deny overrides the user policy to permit. An example would be when the server prevents the updating of a document from someone who is not the original author of that document (even if that person had full update access granted by the record owner). This kind of institutional policy is important for ensuring the integrity and accuracy of data contributed to the PCHR by external healthcare providers. Lastly, when no policies exist on either the institutional or user side to govern a certain action, the institutional side defaults to a lax policy (always allow) while the user side defaults to a strict policy (always deny). Indivo's dual-class (server and individual) approach to access policies is a defining feature of the system. The policy evaluation rules afford maximum control by the record owners over their personal health data within the boundaries set by the system administrators.

The Indivo Server uses the data storage tier to store the various data documents that make up a user's PCHR. In Indivo, the data storage tier is encrypted to protect users even in the event of hardware loss or theft. Encryption keys are hosted on a separate physical server to prevent decryption of patient data if the data storage machine is ever compromised. Further, each record stored in the system is fractured into loosely-related, encrypted data packets to mask the size of an individual record – simply having a large record may be revealing of a complex medical history. The design of the data storage tier is critical, particularly given a recent report of poor data protection in non-server based PHR [7]. Encryption of data at rest also contributes to the long term secure preservation of patient data.

**Figure 2:**
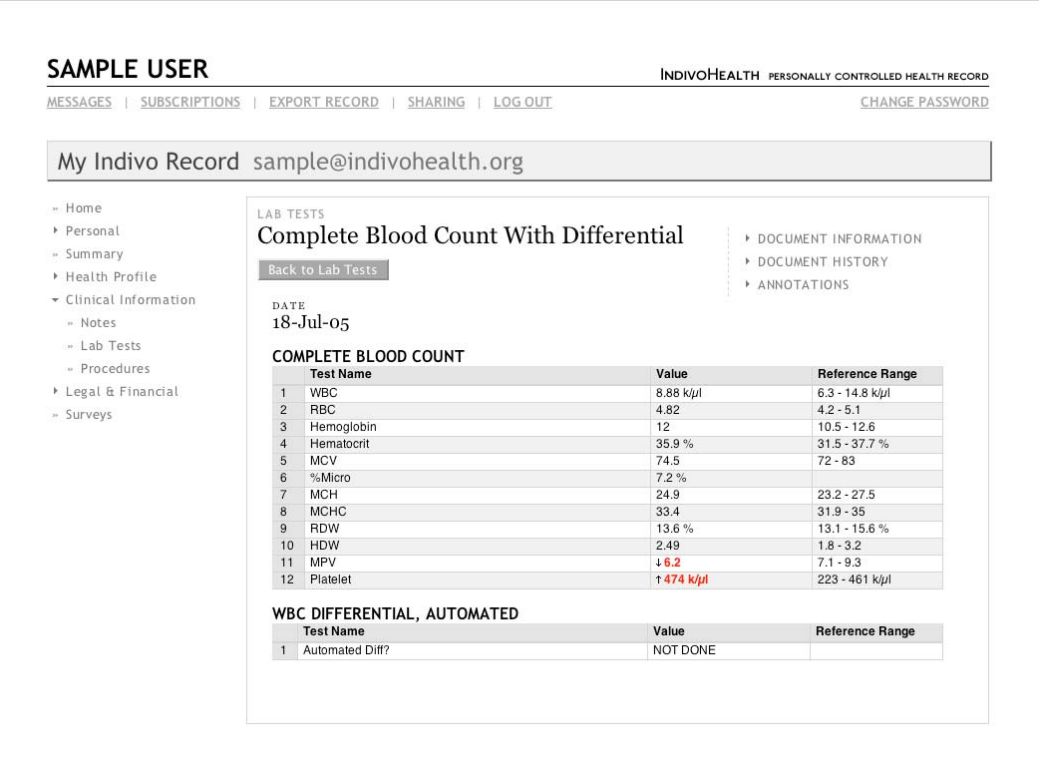
The Indivo user interface showing laboratory test results.

**Figure 3:**
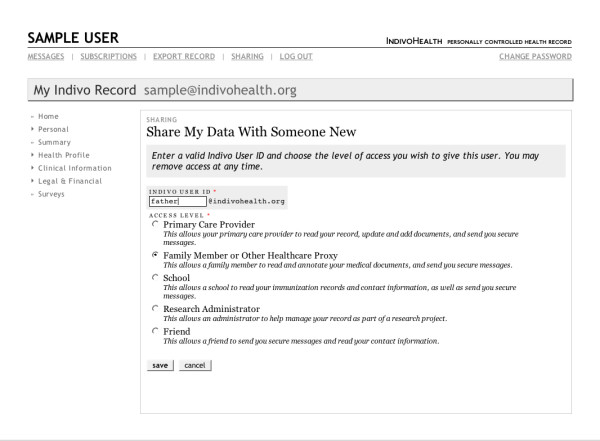
The Indivo user interface showing the options presented to an individual for sharing her record information.

The graphical user interface (GUI), shown in Figures 2 and 3, is responsible for presenting the data contained in the patient's record in a meaningful and comprehensible way. Because the user interface obtains all PCHR data from the Indivo Server, all policies are automatically applied – any action that the user interface tries to perform is passed through the middle tier via the API and thus subject to the security policies that are enforced there. The user interface also helps patients configure the policies that the middle tier will enforce.

Because the user interface interacts with the Indivo Server as a client, via a standard API, a single Indivo Server can support multiple user interfaces depending on the needs of the patient population. The reference implementation of Indivo provides a simple interface for patients, care providers, and administrators using the World Wide Web. The Indivo API is open, allowing others to create their own user interfaces, which can be highly customized in look and feel and provide targeted views of health data for various subsets of the Indivo user community. For example, a tool for managing chronic illnesses like diabetes might only show the user a portion of her record, but may incorporate distinctive workflows and manage specialized data types that are not displayed by the reference implementation's user interface.

By designing the graphical user interface as a client that uses the standard API, the Indivo approach encourages a distributed process of application development that supports creation of "home grown" as well as commercial modules.

### Standards and interoperability

Since the inception of the project in 1998 [8] the Indivo team has been committed to transparency and open standards. All communication with the Indivo system occurs through standard web protocols. The approach to interoperability of PCHRs with electronic medical records (EMRs) and other information systems relies on a simple principle – use widely-adopted, standardized methods for exchanging (importing and exporting) data. Currently, Indivo handles both the Continuity of Care Record (CCR) and the Continuity of Care Document (CCD) for such information transfer and is working closely with the Healthcare Information Technology Standards Panel (HITSP) [9] on interoperability. Standard coding systems, such as LOINC, may be used when the source data provider supports them. Our mechanism for importing data from EMRs and other sources, the subscription agent, is discussed below.

The Indivo architecture is document-centric, with a document model adapted for information needed by patient-centric applications, rather than one which simply wraps electronic health record data. The document model standard is open and available as part of the Indivo open source suite. The highly granular design of the Indivo document model greatly facilitates patient control over exchange of discrete portions of the record. This, in turn, enables individual autonomy in the control of information flows in and out of the PCHR and across a health information exchange.

## Results and discussion

### Health information exchange

By building on the core Indivo system, adding only a few architectural components, we demonstrate how (1) a PCHR is tightly integrated in an information exchange (HIE) model, based on patient ownership and control of their own medical data; and (2) the PCHR becomes a vehicle for communication and decision support.

#### Authentication

In a PCHR-based HIE, actors, such as patients and providers, need to be identified for three tasks – account provisioning, accessing the record, and populating the record with data. The root of trust in healthcare is, and always has been, the patient-physician relationship. Patients are known to their primary care and specialty practices; identification of patients is best accomplished in this setting. The existing web of trust, upon which the healthcare system relies, tends to preclude the sort of wholesale, large scale fraud that might occur in a system that closes the loop on authentication without this human-human interaction required for every new registrant. Hence, we rely on provisioning at physician offices and hospitals (for example, by the registration desk at Children's Hospital Boston), or through well-established identity management systems, such as the certificate-based Kerberos identity management system at the Massachusetts Institute of Technology (which, in turn, relies on the university's network of trust with its students, faculty and staff). At the provisioning event, the patient receives a means for accessing the record. This could involve any of a number of authentication methods including username and password or second factor authentication using, as we have previously recommended, the mobile phone as a vehicle for user authentication [10].

Existing institutional workflows can be leveraged to authenticate a patient prior to issuing an identity federation token, which can be used to link a data source with a PCHR. This token is a cryptographically secure identification of a patient at the institution providing data. It is typically a combination of the patient's ID at the institution with a signed version of that ID. This allows the institution to verify tokens that it generated prior to sending data to the patient's record. Examples of institutional workflows used to provide the patients with their tokens include:

• In person – The patient is required to be physically present with proper forms of identification (e.g. passport, driver's license, birth certificate, etc.) in order for an institutional administrator to issue the token.

• Postal system – A letter addressed to the patient is mailed to the address on file with the institution. The letter contains the token.

• Secure Socket Layer (SSL) Certificate – The patient owns an SSL certificate that is issued by the data provider or by an organization that the data provider trusts. The patient visits a secure web site that validates the SSL certificate and extracts relevant information from it (full name, etc). The patient is then presented with a web form that requests additional information required to generate the token (address, date of birth, medical record number, etc). The token is then generated and presented to the patient.

• Existing health information portals – If the patient has already been issued access to a portion of her medical record through a web portal the portal can also display an identity federation token upon request.

In the Indivo model, the record is populated as the patient acquires a copy of her medical data from each site of care. She accomplishes this by establishing a "subscription" relationship, which necessitates that she be accurately matched with her medical data at the relevant hospitals, clinics, pharmacies, and other sources of medical information. We rely on the data provider to authenticate the patient according to routine procedure and then to issue that patient a unique and secure token associated with the institutional record. The patient uses this token to establish the link between her data at the participating healthcare institution and her Indivo PCHR.

The PCHR HIE approach can leverage current work done at the institutional level to provide patients with direct access to data from electronic health record systems. If a patient has been granted trusted access to a hospital portal [11] via an identity proofing process that is acceptable to the hospital, the portal may provide a feature that allows the user to enter their PCHR server and account information and issue a request for privileges to add documents to the user's PCHR record. Special procedures and transitive trust models are required for accessing data from organizations, like electronic prescription networks, that do not have independent relationships with patients.

### Subscription framework

When we first described populating a PCHR with public health data pertaining to newborn care, [12] we proposed simple data transfers from external sources. Though relatively trivial to load data on a one-time basis from an external source into a PCHR, maintaining that data over a period of time (during which data may be added, removed, or altered within the PCHR system, within an external source, or both) poses challenges. The system requires consistency and transaction management in the face of simultaneous, distributed data operations. The individual PCHR must persistently relate a document to its proprietary source object without jeopardizing portability or patient control. As PCHR technology diffuses, the array of available institutional data sources will magnify the challenge.

Since the PCHR is institutionally neutral, it should not encode excessive domain-specific functionality. Rather the knowledge of data formats in specific systems (EMRs, claims processing systems, laboratory information systems and so forth) remains close to the system, rather than in the central PCHR server. The agent-based data subscription framework mostly eliminates the challenges in maintaining concurrency and consistency and maintains the flexibility and independence of participating healthcare providers. The subscription framework is based on the principle that interoperability is achieved with a standardized import and export from the PCHR. Prior to import, we translate EMR, laboratory and medication data into our document model format that is streamlined for use by a PCHR.

We define a "subscription agent" as software whose purpose is to periodically identify new or changed data within an institution's EMR system and to transfer that data to corresponding documents in a patient's PCHR. During each of its periodic executions, the subscription agent performs the following steps:

• identify PCHR owners that it is responsible for synchronizing.

• for those patients, identify new data or changes to data in the EMR that need to be propagated to the PCHR.

• convert data elements from their internal format into PCHR-specific format.

• transfer newly-formatted data to the PCHR.

• persist the relationship between the internal element and its PCHR counterpart, in case it requires updating at a later date.

The Indivo reference implementation includes a framework for rapid development of custom subscription agents. To this date, we have utilized this framework to write subscription agents that support data transfer from the Children's Hospital Boston EMR (a Cerner system), the Allscripts EMR at MIT Medical, and a generic CCR/CCD format has been designed for import from MinuteClinic, Harvard University Health Services Point and Click System, and from the MA SHARE [13] health information exchange. Subscription agents were also developed for the Office of the National Coordinator of Health Information Technology sponsored National Health Information Network (NHIN) demonstration projects [14], and for a national medication history service deployed via an ePrescribing network. Since each of these data formats is unique, Indivo has adopted a flexible methodology for handling common code sets. Codes in Indivo include references to the formal coding system and version in use.

In order to participate as first class citizens within the PCHR framework, subscription agents must be registered and vetted by the PCHR server administrator. Agents are granted a system role that can be used to customize their permissions within the PCHR ecosystem. In the Indivo Reference implementation, agents are identified by username and authenticated via password. An administrator manually registers the agent with the system, and the agent is then made available to users through the user interface. Vetting the authenticity of the agent is important since PCHR users will be granting the subscription agent permissions to add documents to their record. Those documents will be tracked by the Indivo system and identified as originating from the healthcare data source associated with the subscription agent. Other PCHR systems may choose to allow subscription agents to programmatically register themselves with a "subscription registry" displayed in the user interface. Once the subscription agent is registered, it becomes available for users to actively subscribe to the corresponding data source. The process of subscribing is straight-forward. The user

• selects a data source, such as a hospital, to link to his PCHR,

• acquires identity federation token from data source (as discussed in the previous section),

• locates data source entry in the subscription registry via the PCHR user interface,

• creates/edits/saves subscription configuration (using identity federation token) in PCHR account,

• adjusts access policies to permit the subscription agent to add and update data within the account.

The Indivo GUI streamlines this process. In this instance the final step, adjusting access control policies, is generally done transparently via a preset bundle of policy options. Indivo relies on institutions to choose what data are appropriate to share with patients through PCHRs. For example, some institutions choose not to share physician notes or to provide certain data to patients after a delay [11].

#### Data stewardship

Storing medical data from accredited institutions under patient control requires extreme clarity regarding the patient's rights to their own medical record. Indivo ensures that the patient is in complete control of the PCHR and is allowed to make all decisions regarding the use of the data. This approach does not have any implications for the originating provider's use of the source data provided to the PCHR via the subscription framework. Hospitals are still as free as they have ever been, for instance, to de-identify and sell patient information. The data aggregated in the PCHR "container," however, are the property of the patient. This is a critical concession that the healthcare industry must make if patients are expected to take an active role in coordinating and aggregating their medical information. Indivo does not support even de-identified extracts of data from patient records without explicit individual consent.

The idea of fully patient controlled healthcare information poses legitimate challenges to healthcare providers. Full control implies that the veracity of the information in the record may be suspect. In the Indivo system, the patient has complete control over the sharing and distribution of her record. She is also allowed to annotate any document in the record, update documents that she originally created, and hide, but not delete, documents that are either out of date or that she does not wish to share. Personal control does not, in this case, extend as far as document content, although the user is always free, at any time, to add annotations to any document explaining "the other side of the story." Users can also authorize providers or other users to make annotations on their behalf.

For a PCHR-based health information exchange to be feasible, viewers of these data, especially healthcare providers who make decisions based on what they read, must be confident in an accurate and trustworthy view into the patient's health history. Hence, Indivo limits content modification. The system will not allow the patient to modify a lab test value returned by a hospital system. This is a feature – in return for not allowing the user to edit the information, Indivo presents it to providers and other authorized users as originating from the trusted (medical) source.

With simple controls embedded in the user interface, patients may share clinical data from the Indivo PCHR with another individual or class of individuals. The screen with sharing options is shown in Figure 3. The simplest method for sharing uses an "invite" and gives a specified person time-limited read-only access. A similar action can be taken to give access to all members of, for example, a primary care practice. To make the record, or portions of the record, more widely available, the individual may choose to register the PCHR with a record locator service to enable access by authorized healthcare providers across a health information exchange. We demonstrated this capability at the third NHIN Forum, [14] recognizing that there is a critical tradeoff between tight control and accessibility of the record for medical care.

Indivo also fully supports proxy roles. The adult child of an elderly parent may be the primary decision maker, and may therefore be granted full privileges by that parent. For a deployment at Children's Hospital Boston, we are confronting special considerations around data ownership in the case of mature minors whose rights to consent to medical care vary according to condition, and from state to state. In defining access roles for families with mature minors, the privacy of a minor's information must address the requirements of federal, state and local statutes, institutions, providers' clinical judgment, and the needs of individuals. These issues necessitate careful consideration about both what data the institution exposes to subscription agents, and how family roles are handled by the Indivo system [15].

### Communication

In addition to enabling patient-controlled health information exchange, the Indivo PCHR provides a virtual medical home with modalities for communication among patients, clinicians, researchers and public health authorities.

#### Communication for personal health

The PCHR creates a unique virtual medical home with a direct channel to individuals to engage in health promotion and disease management at home, where people have always used complex strategies to manage their healthcare [16]. A health risk assessment (HRA) survey, for example, can be administered within the patient's own medical context (defined broadly). Unlike in a traditional web-based survey, here the responses to the HRA are stored in the PCHR and immediately available to drive tailored and targeted decision support – potentially in combination with clinical data elements acquired through the subscription service. For example, in a study sponsored by the Centers for Disease Control and Prevention (CDC) Health Protection Research Initiative, we surveyed employees at the Hewlett Packard Corporation, and provided tailored and targeted decision support around influenza prevention and control (appropriate use of vaccination, dangers of "presenteeism", etc). In that deployment, the decision originated locally, but there is also a potentially large market for decision support provided remotely over a service oriented architecture [17,18].

#### Communication for population health

Patients may also share data with public health authorities or researchers. Since each PCHR is an integrated source of an individual's healthcare information among multiple sites of care and over time, a view across PCHRs is a comprehensive view across a population. The population sample obtained through PCHRs is by definition an opt-in sample, since individuals may or may not have PCHRs and may or may not elect to share their information. Hence, there are inherent biases in any sample obtained. We believe strict adherence to the personal control doctrine is preferable to involuntary sharing of "de-identified" data. While the latter may seem attractive at first blush, the approach is shortsighted since the difficulty of truly de-identifying data [19-22] limits the types of data which can be shared and will forfeit rich clinical and genomic data. Rather, the PCHR models for sharing data necessarily rely on either individual altruism [23] or incentivizing of those individuals. Notably, current models for health information exchange also are either opt-in or opt-out, [24] and could produce similar biases depending on the level of trust and participation in the system.

#### Indivo communication components

In addition to the core access controls, four open source components support communication: the messaging module, the survey tool, the decision rules engine, and the broadcast module.

• The messaging module is generically based on a web mail [25] model, with all communication documented and stored in the PCHR.

• The survey tool allows a researcher or public health authority to rapidly create complex surveys with branching logic. The patient receives the survey within the Indivo user interface and while it appears very similar to any web-based survey, all responses are stored in the PCHR for access by another application – a decision rules engine – which can take actions based on rules. Also, the responses are easily exported in XML which is readily converted to a standard format for analysis, such as SAS. We have implemented the decision engine both locally and as a web service.

• The broadcast module, recently described in the literature [26] is under development. Briefly, the module is for communication with members of a research cohort, who can choose to "tune in" to select types of information, like research study results that pertain to them. For example, if research subjects have an Indivo PCHR storing their genomic information [22] they can choose to receive information pertaining to particular single nucleotide polymorphisms that they may have. Since the broadcast is general and the patients choose whether or not to tune in, this approach allows researchers to have access to only de-identified data (as is often required by institutional review boards) while still providing patients with tailored and targeted communication.

### Indivo deployments

There is a fundamental challenge in demonstrating the utility of a ubiquitous interoperable PCHR *before *the environment supports either ubiquity or wide-scale interoperability. Hence, the Indivo deployments to date have tended to focus on demonstration and evaluation of key features of PCHRs (Table 1). The current round of research focuses on the use of PCHRs for healthcare interventions. For example, we and colleagues at Children's Hospital Boston are deploying a workplace health promotion study at the Massachusetts Institute of Technology. Also, researchers at McMaster University have developed an Indivo PCHR application, MyOscar, that not only fully utilizes the Indivo API, but is built using the Indivo code base (licensed under the GNU Lesser General Public License (LGPL) [27]. Researchers at NTIU in Norway have also explored the application of Indivo for the Norwegian health system [28].

**Table 1:** Indivo deployment

**Year**	**Deployment**	**Functionality**
2001	Emergency department follow-up	Emergency department patients with possible streptococcal throat infections given PCHRs and provided notification and decision support around throat culture results.
2004-	Networked primary care PCHR	The Canadian National Research Council and a team at McMaster University used the Indivo source code to develop a series of applications around a networked primary care PCHR, including interaction with an open source EMR [29] and a pharmacy system.
2005	Worksite employee health program	After consenting to participate in a clinical trial, employees at Hewlett Packard were randomized and given an Indivo PCHR. Through HRA surveys, a risk category for influenza was assessed. The decision rules engine sent tailored and targeted messages to individuals regarding influenza prevention and control (appropriate use of vaccination, dangers of presenteeism, etc) This deployment fully tested the three tiered architecture and the messaging module, survey module and decision rules engine.
2007	Office of the National Coordinator of Health Information Technology NHIN demonstration	Regional and interregional sharing of medication and registration data compliant with the Connecting for Health Common Framework for health information exchange [14, 30]. Registration of PCHRs with a regional record locator service and communication across geographically diverse Regional Health Information Organizations using common protocols. Data exchange between Massachusetts SHARE and the Indiana Health Information Exchanged was fully prototyped and demonstrated.
2007-	Access control test application	Researchers at the Norwegian University of Science and Technology are implementing an Indivo testbed for studying the complex technical issues around access control.
2007-	Immunization decision support	The Indivo PCHR under patient control, will send a de-identified clinical extract to the immunization registry forecasting module at the Massachusetts Department of Public Health. The forecasting module returns recommendations for next immunizations due, activating patients to help primary care providers ensure that their children have up to date vaccine status.
2007-	Pediatric teaching hospital	Full scale deployment of a PCHR at Children's Hospital Boston with subscription agent functionality, information sharing with public health, research and schools. Demonstrating subscription, access control, decision support, results broadcast.
2007-	Employee health program at a medical health maintenance organization serving a university population	Scale-up of the Hewlett Packard employee health program to the student and employee population of MIT Medical including a networked PCHR with subscriptions to EMR data and a robust authentication mechanism.
2007	Regional data exchange	CCR/CCD based data exchange between electronic health records and Indivo.

## Conclusion

The recent release of Indivo version 3.0 included improvements in usability, performance (server and client), and architecture over our previous software versions (PING 1.x and 2.x). While we will continue to support, improve, and issue further software releases based on our 3.0 technology, we also have conceptual improvements in store for a 4.0 release. Specifically, we will (1) adopt the concept of a health URL for every Indivo user; (2) provide a mechanism for users and agents in the system to send requests to other users for access privileges on their records; (3) leverage the latest enterprise technologies to further improve performance and scalability; and (4) decentralize certain aspects of the user interface by modifying our API to more closely model today's popular web platforms such as Flickr and Amazon. Our current Indivo deployments are focused on proving the utility of the PCHR as a platform for patient centric integration of healthcare information and for the development of effective, patient driven applications to improve the quality, effectiveness, and convenience of the healthcare system. A widespread PCHR platform, built on fully open APIs and standards, may, by providing aggregated data and patient-led security, enable the development of an ecosystem of personalized healthcare applications. Unlike other approaches to healthcare information exchange and aggregation, the PCHR model fully empowers users to identify new uses for the healthcare data, and supports data providers' participation by allowing them the choice of different levels of engagement with the data provisioning process.

## Availability and requirements

• **Project name: **Indivo

• **Project home page: **

• **Operating system(s): **Platform independent

• **Programming language: **Java, PHP

• **Other requirements: **Java 1.5 or higher, Tomcat 4.0 or higher, PHP 5.2 or higher with PEAR DB, Log, and Config packages, Apache Web Server 2.0, MySQL, PHP-Java Bridge 4.1.2 or higher

• **License: **LGPL.

• **Any restrictions to use by non-academics: **None

## Competing interests

The authors have provided unpaid consultation to the Dossia founders.

## Authors' contributions

KDM, WWS, WCRC, and JMA all contributed to drafting of the manuscript and formulation of the framework.

## Pre-publication history

The pre-publication history for this paper can be accessed here:


